# Supplementary material for: The Relationship between Vitamin D Metabolites and Androgens in Women with Polycystic Ovary Syndrome
Source: Nutrients. 2020 Apr 26;12(5):1219. doi: 10.3390/nu12051219 (PMC7282251; doi:10.3390/nu12051219)
Supplement: Supplementary file 1 [file nutrients-12-01219-s001.pdf]

Table 1. Pearson's correlations between markers of vitamin D status, androgens and features of polycystic ovary syndrome.

	Body Fat (%)	HOMA-IR #	hs-CRP (mg/L) #
**Total 25(OH)D (nmol/L)**	-0.139 (0.381)	**-0.310 (0.046)**	-0.231 (0.146)
**Free 25(OH)D (pmol/L)**	**-0.502 (0.001)**	**-0.582 (<0.001)**	**-0.512 (0.001)**
**Bioavailable 25(OH)D (nmol/L)**	-0.113 (0.493)	0.152 (0.355)	0.106 (0.526)
**Vitamin D Binding Protein (ug/mL)**	0.251 (0.113)	0.160 (0.319)	**0.348 (0.028)**
**Testosterone (nmol/L)**	-0.004 (0.980)	-0.022 (0.898)	-0.075 (0.635)
**SHBG (nmol/L)**	-0.101 (0.514)	-0.159 (0.348)	0.068 (0.665)
**FAI #**	0.045 (0.772)	0.063 (0.712)	-0.068 (0.666)

Correlation coefficient (P-value). #: Spearman's correlations. 25-hydroxyvitamin D: 25(OH)D; The homeostatic model assessment of insulin resistance: HOMA-IR; High-sensitivity C-reactive protein: hs-CRP; Sex Hormone-Binding Globulin: SHBG; Free Androgen Index: FAI. Bold values indicate statistical significance.

Table 2. Multivariable linear regression between markers of vitamin D status and androgens.

	Total 25(OH)D (nmol/L)	Free 25(OH)D (pmol/L)	Bioavailable 25(OH)D (nmol/L)	Vitamin D Binding Protein (ug/mL)
**Model 1**				
**Testosterone (nmol/L)**	0.002 (-0.010, 0.013)	-0.009 (-0.048, 0.029)	0.024 (-0.140, 0.188)	0.001 (-0.0005, 0.002)
**SHBG (nmol/L)**	0.300 (-0.026, 0.626)	0.723 (-0.584, 2.031)	-0.964 (-5.986, 4.058)	-0.010 (-0.049, 0.030)
**FAI**	-0.002 (-0.007, 0.003)	-0.007 (-0.026, 0.011)	0.013 (-0.059, 0.085)	0.0003 (-0.0003, 0.001)
**Model 2**				
**Testosterone (nmol/L)**	0.008 (-0.003, 0.018)	-0.020 (-0.066, 0.025)	0.001 (-0.147, 0.149)	0.001 (-0.0003, 0.002)
**SHBG (nmol/L)**	**0.369 (0.021, 0.716)**	0.795 (-0.774, 2.363)	-1.275 (-6.427, 3.877)	-0.005 (-0.049, 0.038)
**FAI**	-0.002 (-0.007, 0.004)	-0.008 (-0.030, 0.015)	0.012 (-0.061, 0.086)	0.0003 (-0.0003, 0.001)
**Model 3**				
**Testosterone (nmol/L)**	0.008 (-0.005, 0.020)	-0.026 (-0.081, 0.030)	0.095 (-0.076, 0.267)	0.001 (-0.0004, 0.002)
**SHBG (nmol/L)**	0.405 (-0.007, 0.818)	1.114 (-0.872, 3.099)	-1.297 (-7.583, 4.989)	-0.010 (-0.060, 0.039)
**FAI**	-0.003 (-0.009, 0.004)	-0.014 (-0.043, 0.015)	0.045 (-0.045, 0.135)	0.0003 (-0.0004, 0.001)

Values are unstandardized beta-coefficients (95% confidence intervals). Model 1 is adjusted for age. Model 2 is adjusted Model 1 plus body fat percentage. Model 3 is adjusted for Model 2 plus the homeostatic model assessment of insulin resistance (HOMA-IR). Free androgen index was log transformed. 25-hydroxyvitamin D: 25(OH)D; Sex Hormone-Binding Globulin: SHBG; Free Androgen Index: FAI. Bold values indicate statistical significance.
